# A tetraploidization event shaped the *Aquilaria sinensis* genome and contributed to the ability of sesquiterpenes synthesis

**DOI:** 10.1186/s12864-021-07965-9

**Published:** 2021-09-07

**Authors:** Fanbo Meng, Tianzhe Chu, Qiang Tang, Wei Chen

**Affiliations:** 1 State Key Laboratory of Southwestern Chinese Medicine Resources, Chengdu University of Traditional Chinese Medicine, 611137 Chengdu, China; 2 Innovative Institute of Chinese Medicine and Pharmacy, Chengdu University of Traditional Chinese Medicine, 611137 Chengdu, China; 3 School of Pharmacy, Chengdu University of Traditional Chinese Medicine, 611137 Chengdu, China; 4 School of Basic Medical Sciences, Chengdu University of Traditional Chinese Medicine, 611137 Chengdu, China; 5 School of Life Sciences, North China University of Science and Technology, 063210 Tangshan, China

**Keywords:** *Aquilaria sinensis*, Tetraploidization, Collinearity, Transcriptome, Sesquiterpenoid biosynthesis

## Abstract

**Background:**

Agarwood, generated from the *Aquilaria sinensis*, has high economic and medicinal value. Although its genome has been sequenced, the ploidy of *A. sinensis* paleopolyploid remains unclear. Moreover, the expression changes of genes associated with agarwood formation were not analyzed either.

**Results:**

In the present work, we reanalyzed the genome of *A. sinensis* and found that it experienced a recent tetraploidization event ~ 63–71 million years ago (Mya). The results also demonstrated that the *A. sinensis* genome had suffered extensive gene deletion or relocation after the tetraploidization event, and exhibited accelerated evolutionary rates. At the same time, an alignment of homologous genes related to different events of polyploidization and speciation were generated as well, which provides an important comparative genomics resource for Thymelaeaceae and related families. Interestingly, the expression changes of genes related to sesquiterpene synthesis in wounded stems of *A. sinensis* were also observed. Further analysis demonstrated that polyploidization promotes the functional differentiation of the key genes in the sesquiterpene synthesis pathway.

**Conclusions:**

By reanalyzing its genome, we found that the tetraploidization event shaped the *A. sinensis* genome and contributed to the ability of sesquiterpenes synthesis. We hope that these results will facilitate our understanding of the evolution of *A. sinensis* and the function of genes involved in agarwood formation.

**Supplementary Information:**

The online version contains supplementary material available at 10.1186/s12864-021-07965-9.

## Background

*Aquilaria sinensis* (2n = 16) is the species of the genus *Aquilaria* of Thymelaeaceae. It grows mainly in tropical climates and is an economically important medical plant. Once wounded or infected by fungi, *A. sinensis* can produce a precious natural flavor product called agarwood[1], which is widely used in traditional Chinese medicine, handicrafts and religious ceremonies worldwide[1–4]. Due to the limited natural production and increasing demand of agarwood, the *A. sinensis* species are on the verge of extinction. Therefore, clarifying the evolution of *A. sinensis* and the mechanism of agarwood synthesis will be helpful for the protection of *A. sinensis* and will also improve the production of agarwood.

Whole-genome duplication (WGD) or polyploidy, followed by gene loss and diploidization, is an important evolutionary force for many organisms, which not only contributes to their origin and diversification, but also facilitates their adaptation to environmental changes[5–8]. In particular, recursive polyploidization, polyploidization of different ploidy, and genome rearrangement have been observed in angiosperms[9, 10]. As a valuable plant, the genome of *A. sinensis* has been sequenced by two independent groups[11, 12]. It was reported that the genome of *A. sinensis* experienced a recent WGD event[11]. However, due to the lack of in-depth exploration, the ancestral ploidy of *A. sinensis* remains unclear.

Recent studies also demonstrated that agarwood contains many chemical components with different pharmacological activities, such as sesquiterpenes, diterpenes, and chromones[13–16]. Therefore, based on the transcriptome data, the expression profiles of genes related to the sesquiterpene synthesis or stress responses during the formation of agarwood were analyzed[17–19]. Accordingly, the hypothesis that agarwood is the product of plant defense reaction was proposed[17]. Since no reference genome was available then, it was impossible to analyze the genes participated in the entire regulatory pathway of sesquiterpene synthesis. By combining the genome and transcriptome data of *A. sinensis*, the complete set of genes participated in sesquiterpenoid production, plant defense and agarwood production were analyzed in a recent work[12]. It was found that those genes participate in the biosynthesis of sesquiterpenoids via the mevalonic acid (MVA), 1-deoxy-D-xylulose-5-phosphate (DOXP), and methylerythritol phosphate (MEP) pathways. However, the expression changes of genes involved in sesquiterpene biosynthesis during the formation of agarwood have not been analyzed.

Keeping these in mind, in the present work, by using our previously developed pipeline for decoding complex genomes[20, 21], we reanalyzed the *A. sinensis* genome by selecting the grape (*Vitis vinifera*) and cacao (*Theobroma cacao*) genomes as the references, and inferred the ploidy of *A. sinensis* paleopolyploid and its occurrence time. By performing the collinearity analysis, an alignment of paralogous and orthologous genes among the three genomes was generated. In order to understand the molecular mechanism of agarwood formation after injury to the stem of *A. sinensis*, the expression changes of genes involved in the sesquiterpene synthesis pathway were analyzed. It was found that the polyploidy events promoted the functional differentiation of sesquiterpene synthesis genes in *A. sinensis*.

## Results

### Homologous Gene Collinearity

Identification of gene collinearity regions is the basis for deciphering genome structure and elucidating genome evolutionary history. By using ColinearScan[22], we inferred colinear genes within each genome and those between *A. sinensis* and the two reference genomes, namely cacao or grape (Additional file 1: Tables S1 and S2). To identify paralogous genes in colinearity within the *A. sinensis* genome, we found 1,120, 155, 52, and 13 syntenic blocks containing at least 4, 10, 20, and 50 colinear gene pairs. The total number of gene pairs in these blocks are 8,092, 3,574, 2,223, and 1,084, respectively, with the average of 7.22, 23.06, 42.75 and 83.38 gene pairs per block.

The cross-genome homologous blocks, especially those between *A. sinensis* and cacao, are generally longer than those within the *A. sinensis* genome. For example, the genes from *A. sinensis* and cacao form 27,462 colinear gene pairs located in 2,426 homologous blocks with more than four colinear gene pairs, involving 16,525 (56.6 %) and 15,086 (70.3 %) genes from the two genomes, respectively.

### Evidence for A Paleo-Tetraploidization Event

**Fig. 1:**
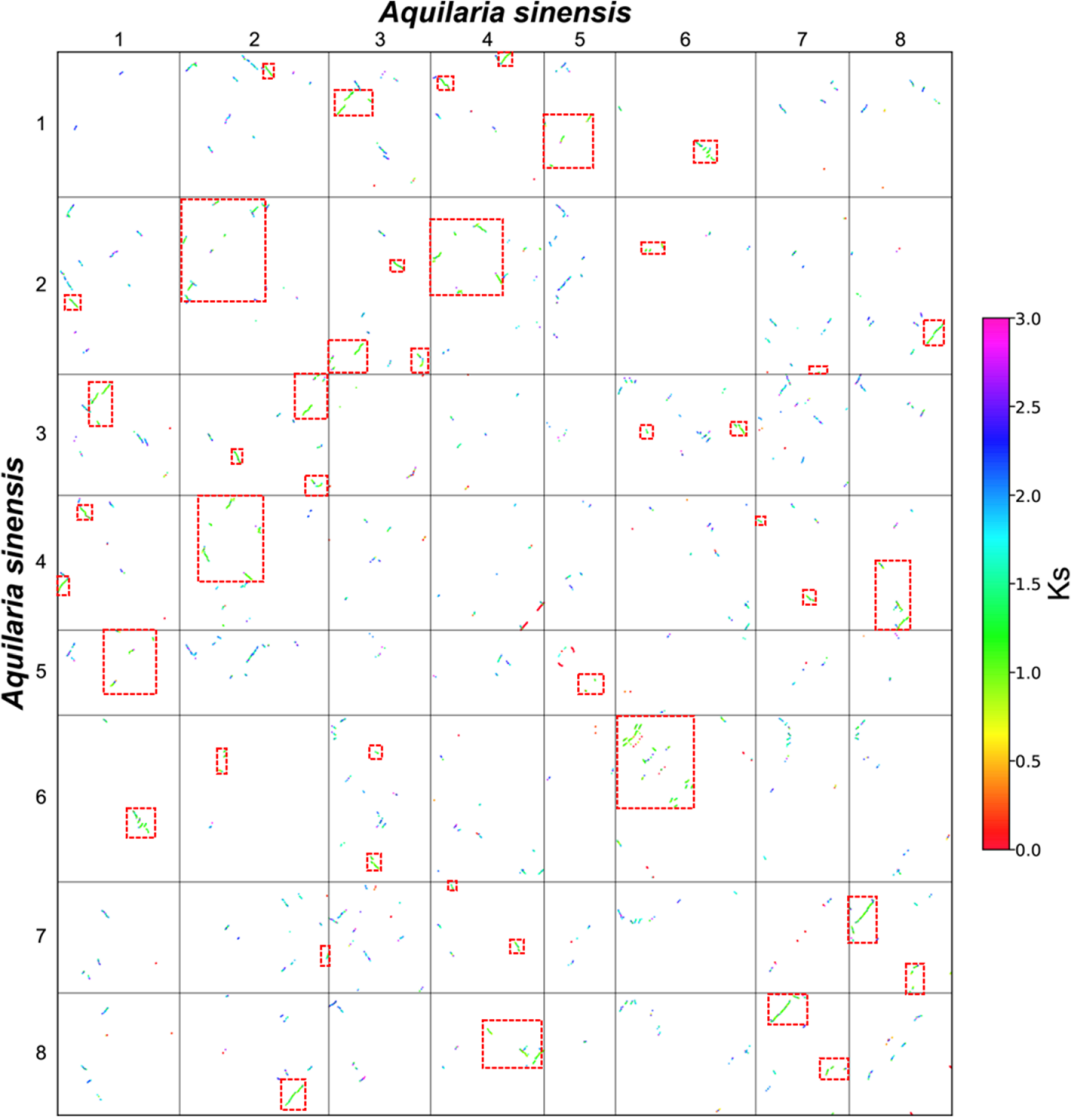
Homologous Genes Dotplot Within *A. sinensis* Genome. ${K}_{S}$ value for homologous genes in each inferred colinear block is shown. Paralogous blocks were identified by red dashed rectangles

Although a recent whole-genome duplication event has been reported in *A. sinensis* genome [11], the ploidy of its polyploidization remains unclear. To clarify this point, we firstly characterized the synonymous nucleotide substitutions on synonymous substitution sites (${K}_{S}$) between the above inferred collinear genes. Then, we constructed a dotplot of homologous genes within the *A. sinensis* genome, in which the collinear genes were displayed by different colors according to their ${K}_{S}$ values (Fig. 1). If there was no further polyploidization after the shared hexaploidization (i.e. the ECH), each genomic region would have two best matching regions (paralogous, with a smaller median ${K}_{S}$) within the *A. sinensis* genome. However, we found that there is only one best matching region in *A. sinensis* (Fig. 1), indicating that it experienced a tetraploidization. We called it as an *A. sinensis*-specific tetraploidization (AST).

**Fig. 2:**
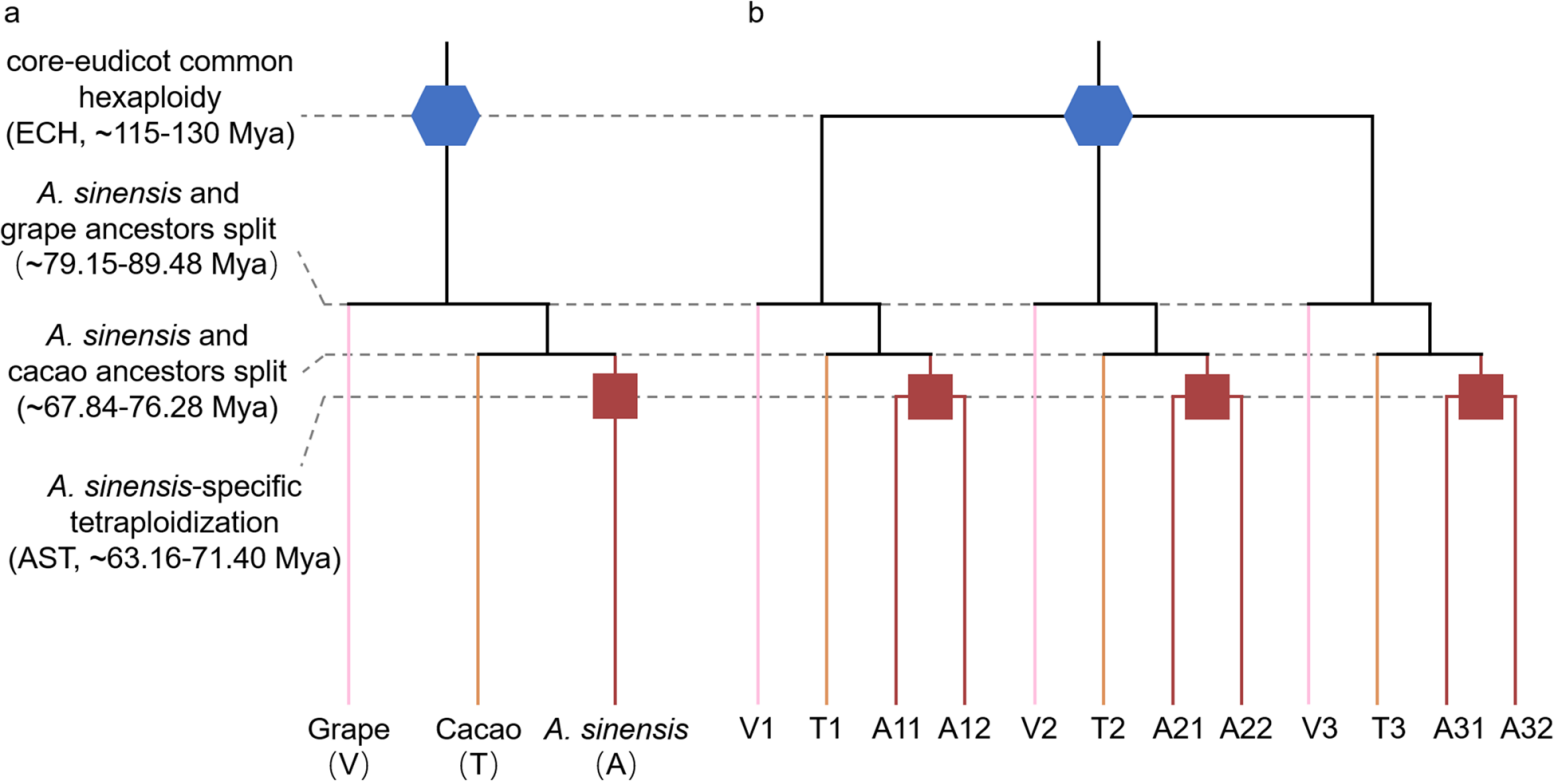
Species and Gene Phylogenetic Trees. **a** Phylogenetic tree of grape (V), cacao (T), and *A. sinensis* (A). ECH is denoted by blue hexagon and the *A. sinensis* paleotetraploidization event is denoted by darkred quadrilateral. **b** Gene phylogeny. Three paralogous genes in the grape and cacao genomes are denoted by V1, V2, V3 and T1, T2, T3, which were generated by the ECH. Each of them has two orthologs and four outparalogs in the *A. sinensis* genome. For example, V1 has two orthologs A11, A12 and four outparalogs A21, A22, A31, and A32 in *A. sinensis*

In addition, by plotting the homologous dotplots between *A. sinensis* and grape genomes, we found that there are two best matching regions (orthologous, with a smaller median ${K}_{S}$) in *A. sinensis*, corresponding to each grape chromosome (Additional file 2: Figure S1). For example, for grape chromosome 2, 15, and 16, which are homologous chromosomes produced by ECH[23], each has two best matching regions in the *A. sinensis* genome. Furthermore, we also drew the homologous dotplots between *A. sinensis* and cacao (Additional file 2: Figure S2), which is more closely related to *A. sinensis* than grape and has not been affected by other polyploidizations after the ECH. It was found that each cacao genomic region has two orthologous regions in the *A. sinensis* genome, which is larger than that between grape and *A. sinensis* genomes. These intergenomic analyses demonstrate that *A. sinensis* experienced a tetraploidization event after the ECH (Fig. 2).

### Event-Related Genomic Homology

According to the above mentioned homologous gene dotplot within the *A. sinensis* genome, we screened the blocks with more than 4 gene pairs based on the ${K}_{S}$ of the collinearity gene pairs and located them to distinguish event-related genome homology regions (Fig. 1). By doing so, the blocks were separated into two types. One type is related to ECH (with a bigger median ${K}_{S}$) (Additional file 1: Table S3), and the other one related to AST (with a smaller median ${K}_{S}$) (Additional file 1: Table S4). Based on their mutual positions in the dotplot, we divided the ECH-related blocks containing genes from different subgenomes into two groups (Additional file 1: Table S4). Similarly, the blocks in grape and cacao were also filtered and divided into two groups (Additional file 1: Tables S5 and S6). In grape, we found a total of 123 blocks related to the ECH event, forming 2314 paralogous pairs, containing 3,978 genes. In cacao, 3108 paralogous gene pairs containing 4,987 genes in 102 collinear regions were found. In *A. sinensis*, there are 178 blocks related to the ECH event, forming 1624 gene pairs, containing 2,570 genes, and for the AST event, there are 86 blocks, forming 1,778 gene pairs containing 3,023 genes (Table 1).

**Table 1:** Number of Duplicated Genes Within Selected Genomes Related to ECH and AST

Species	ECH-related ^a^	AST -related ^b^
*V. vinifera*	123/2314/3978 ^c^	-
*T. cacao*	102/3108/4987	-
*A. sinensis*	178/1624/2570	87/1778/3023

^a^Core eudicot-common hexaploidy (ECH); ^b^Aquilaria-specific tetraploidization; ^c^numbers of blocks/gene pairs/gene numbers

Furthermore, we screened the collinearity blocks related to species divergence events between every two genomes (Additional file 1: Table S7), and found that the gene collinearity between genomes is better than that within genomes. For example, 11,201 (38.36 %) genes from *A. sinensis* could find orthologs in the grape, forming 11,398 orthologous pairs and 486 collinearity blocks.

### Multiple Genome Alignment

**Fig. 3:**
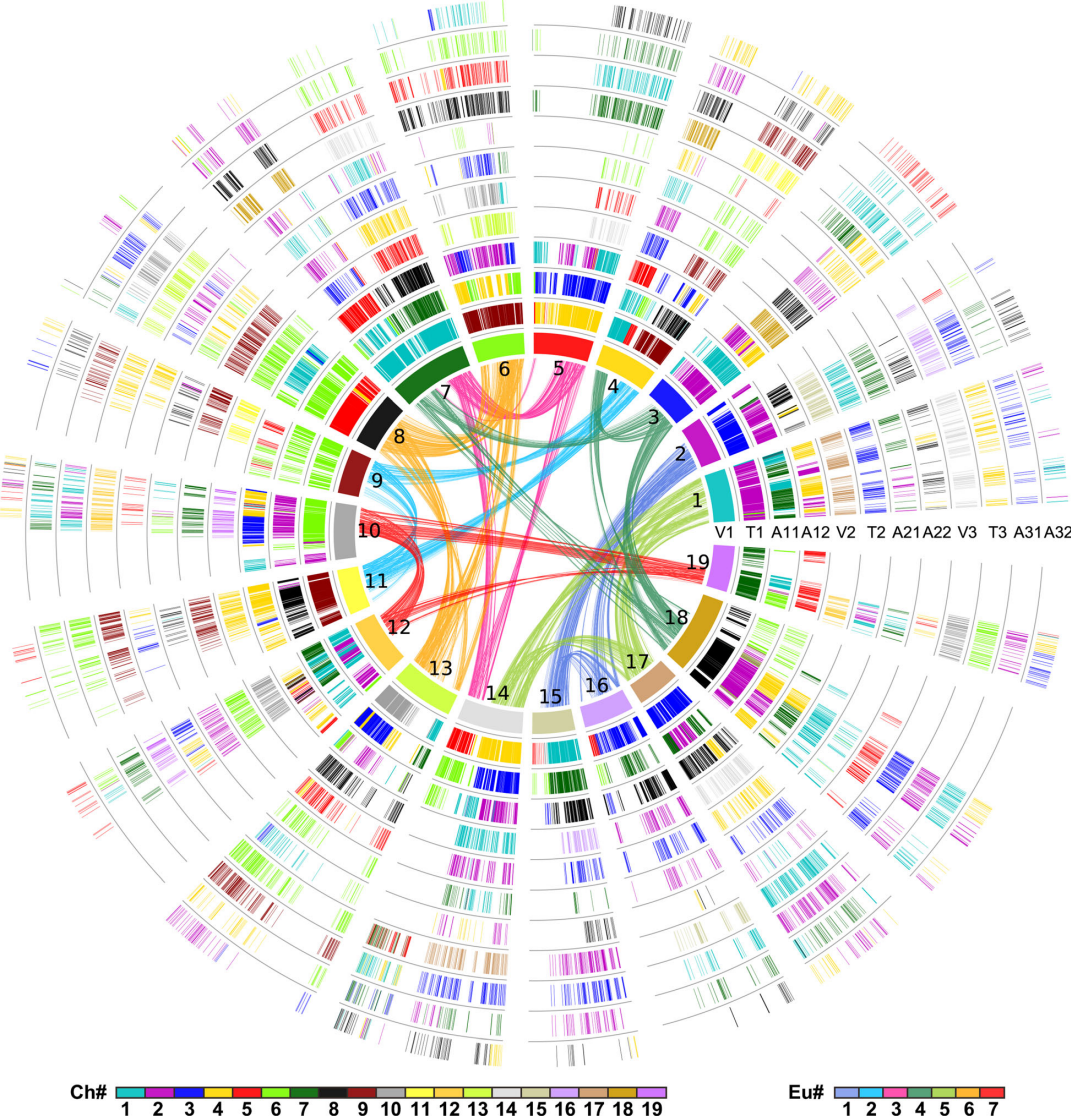
Homologous Alignments of Grape, Cacao, and *A. sinensis* Genomes. The 12 circles show the homology alignment of the homologous regions among grape (V), cacao (T), and *A. sinensis* (A) genomes, with the grape as a reference genome. The curved lines within the inner circle are colored according to the 7 ancestral eudicot (Eu) chromosomes and link the paralogous pairs on the 19 grape chromosomes generated by the ECH. Each circle is formed by short vertical lines that denote homologous genes, which are colored to indicate chromosome number in their respective source plant as shown in the color scheme at the bottom

By using the grape genome as a reference and filling collinear gene IDs into the polyploid event-related colinear gene table (Additional file 1: Table S8), we performed hierarchical and event-related multiple-genome alignments (grape, cacao, and *A. sinensis*) and constructed the homologous gene table. The homologous genes from the three species were arranged in 12 columns, including three columns corresponding to paralogous chromosomes of grape, three columns for paralogous chromosomes of cacao, and six columns cumulatively resulting from the ECH followed by AST of *A. sinensis*. The six *A. sinensis* columns can be classified into three groups corresponding to the hexaploidization-derived chromosomes. Each group contains two columns (corresponding to AST) which is orthologous to a single grape or cacao column, respectively. The table contains paralogous genes in each genome and outparalogs between genomes with each polyploidization, and orthologs between genomes with their ancestral speciation. In order to clearly display the alignment between the three genomes, we converted the homologous collinearity table into a graph (Fig. 3). In Fig. 3, each circle represents a column in the table, and the short lines in the circle represent collinearity genes. In addition, to give a close view of genomic fractionation, a linear graph was used to display a part of the genome-level alignment (Additional file 2: Figure S3).

Since genes specific to *A. sinensis* and absent from the grape couldn’t be represented by using the grape genome as reference, we also constructed a genomic homology table by using cacao as reference (Additional file 1: Table S9). The table thus obtained supported the paleotetraploidization and represented gene collinearity in *A. sinensis*. For example, when cacao was used as a reference, *As1g02078* and *As5g01003* were displayed in the table as collinear genes produced by AST. However, they could not be displayed when grape was used as a reference.

### Genomic Fractionation

The above results indicate that many genes were lost and translocated in the *A. sinensis* genome after experiencing the tetraploidization event. Therefore, we calculated the gene retention or removal rate in the *A. sinensis* genome on each reference chromosome. The chromosomal regions duplicated by the AST often have divergent gene retention levels (Additional file 2: Figures S4 and S5). Regardless of which genome (grape or cacao) was used as the reference, they all showed a lower collinear gene corresponding with *A. sinensis*. In the two sets of orthologous regions, the loss rate of collinear genes relative to different grape chromosomes ranged from 63.80 to 87.52 % (Additional file 1: Table S10). Approximately 81.28 and 87.52 % of the genes in grape chromosome 9 did not have collinear genes in one of the two groups of *A. sinensis* orthologous regions, and 82.93 % of genes did not have correspondence in all homologous regions. The gene loss rates relative to different cacao chromosomes is lower, which ranges from 54.81 to 80.28 % in each of the two sets of orthologous regions (Additional file 1: Table S11). These results indicate that extensive gene deletion or relocation occurred in the *A. sinensis* genome after the AST event.

Furthermore, by using grape and cacao as reference genomes, we characterized the process of successively removing genes in *A. sinensis*. Excluding the loss of chromosome fragments, the number of removing genes was no greater than 15 consecutive genes in most gene deletion cases. A statistical fitness regression showed that deletion patterns followed a near geometric distribution (Additional file 2: Figures S6 and S7, Additional file 1: Table S12). The extension parameters of geometric distributions were 0.3665 and 0.4201 (goodness of fit F-test *P*-values were 0.8848 and 0.9115 to accept fitness) by using grape and cacao as references, respectively (Additional file 1: Table S13).

### Evolutionary Divergence and Dating

**Fig. 4:**
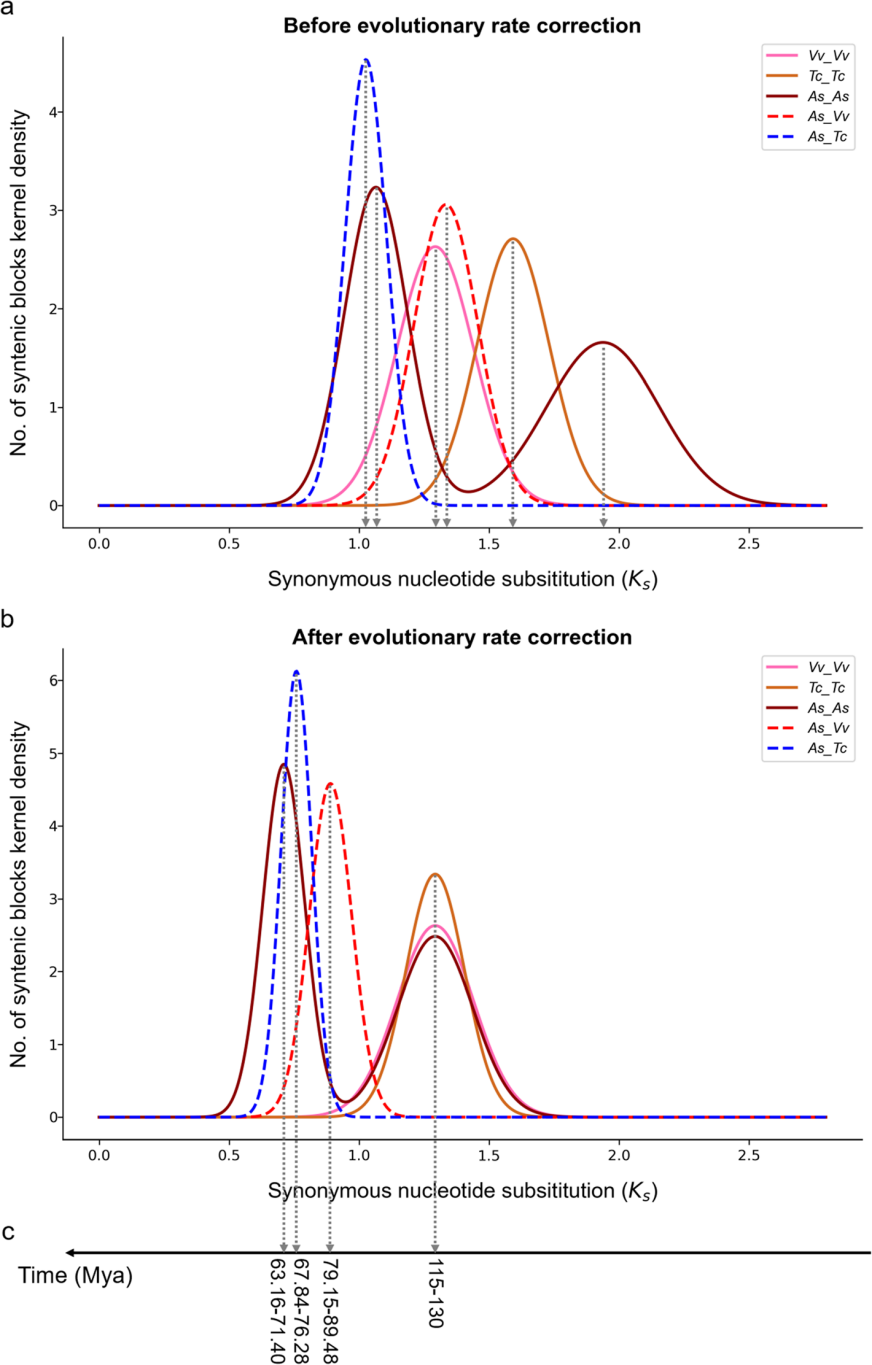
Dating Evolutionary Events. **a** Distribution of original median ${K}_{S}$ between collinear gene pairs in intergenomic blocks (solid curves) and intragenomic blocks (dashed curves). **b **Distribution of adjusted median ${K}_{S}$ between collinear gene pairs in intergenomic blocks (solid curves) and intragenomic blocks (dashed curves). **c** Inferred time

By calculating ${K}_{S}$ on synonymous nucleotide sites within each genome and among different genomes, we estimated the occurrence time of the AST and other key events. As the ${K}_{S}$ distribution between paralogs or orthologs is related to specific events, the normal distribution function was used to perform fitting[21, 24, 25]. We statistically determined the location of its mean (or peak) and variance (Fig. 4a, Additional file 2: Figure S8, and Additional file 1: Table S14). It was found that the ECH-related ${K}_{S}$ peaks from the three genomes are in different positions. The ${K}_{S}$ of grape is 1.2927 (+/- 0.1477), the ${K}_{S}$ of cacao is 1.5922 (+/- 0.1383), and the ${K}_{S}$ of *A. sinensis* is 1.9379 (+/- 0.3066). These values indicate that the evolution rate of grape is the slowest, and the evolution rates of cacao and *A. sinensis* are 23.17 % ((1.5922–1.2927)/ 1.2927) and 49.91 % ((1.9379–1.2927)/ 1.2927) faster than that of grape, respectively.

In order to adjust the evolution rate corresponding to ECH events, we aligned the peaks in different genomes to the same position (Fig. 4b, Additional file 1: Table S15) by using the method proposed by Wang et al.[20, 21]. Since the ECH occurred ~ 115–130 million years ago (Mya)[23, 26], we inferred that the AST event occurred ~ 63.16–71.40 Mya. In addition, we inferred that ancestors of *A. sinensis* and grape split at ~ 79.15–89.48 Mya, and ancestors of *A. sinensis* and cacao split at ~ 67.84–76.28 Mya (Fig. 4c).

### Sesquiterpenoid Biosynthesis Related Genes

**Fig. 5:**
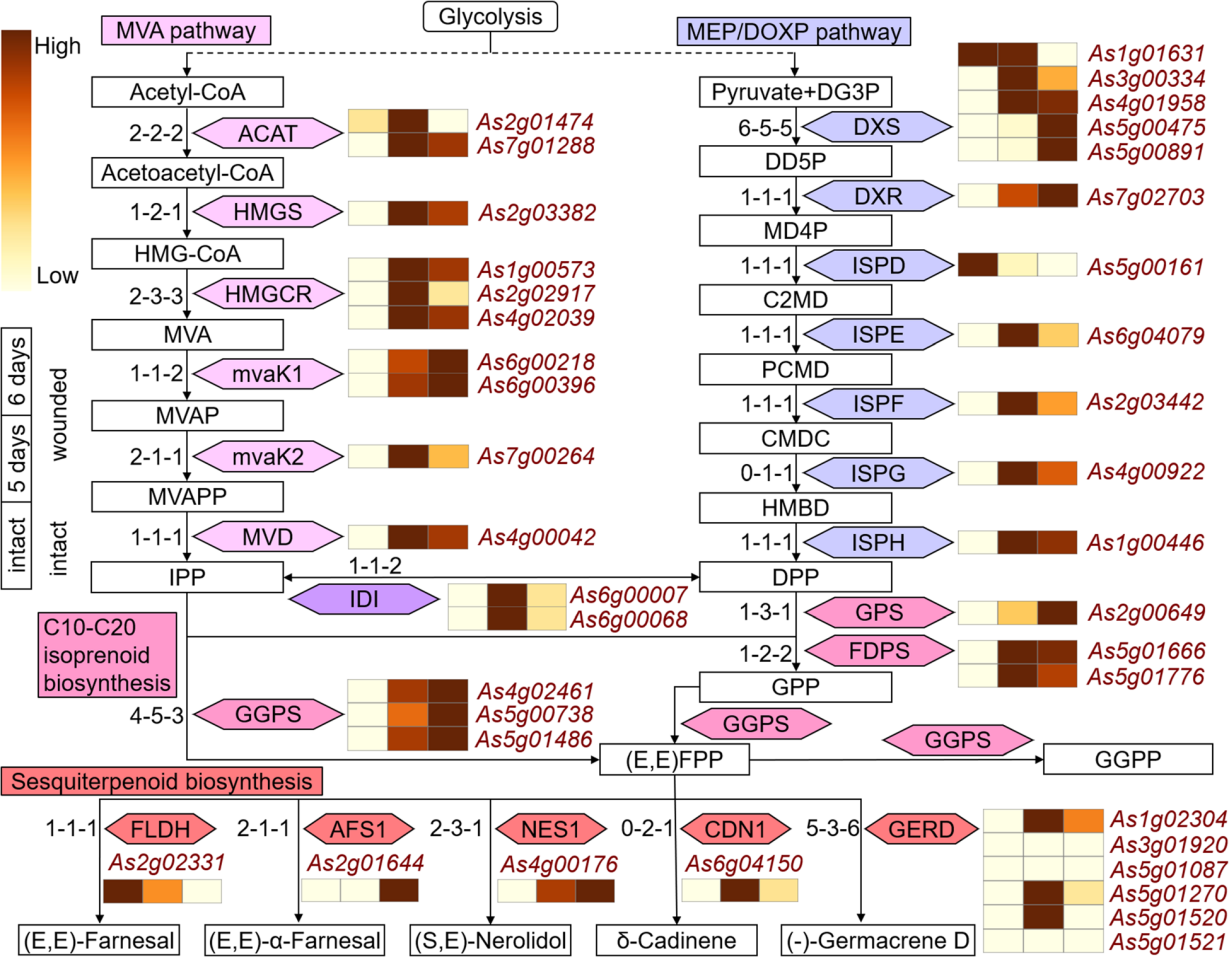
Inferred Sesquiterpene Biosynthesis Related Genes and Their Expression Profiles in the Stem of *A. sinensis* at Three Different States. The sesquiterpenoid biosynthesis related genes in grape, cacao, and *A. sinensis*, including genes in the mevalonic acid (MVA) pathway, 1-deoxy-D-xylulose-5-phosphate (DOXP) or methylerythritol phosphate (MEP) pathway, and sesquiterpenoid biosynthesis. The notation ‘1-1-1’ indicates that one gene was identified in grape, cacao, and A. sinensis, respectively. Gene expression level in the three different states (intact, wounded 5 days, and wounded 5 + 1 days) of *A. sinensis* stems was characterized by the TPM value. The dark red color indicates high expression levels in different states

In order to explore the copy status of genes related to sesquiterpene synthesis in *A. sinensis* and reference genomes, we used KAAS to annotate genes in the three genomes. Based on the KEGG database, the genes participating in sesquiterpenes synthesis were obtained, including genes in the terpene backbone synthesis pathways (MVA, MEP/DOXP pathway) and the sesquiterpene synthesis. Almost every node in the above mentioned pathway has one or more gene copies among the three species (Fig. 5, Additional file 1: Table S16). A total of 39 genes have been annotated in *A. sinensis*, including six (-)-germacrene D synthase (GERD) genes. Compared with the MVA pathway (only Acetyl-CoA (ACAT) and diphosphomevalonate decarboxylase (MVD) had the same number of gene copy in three genome), the number of gene copies in the MEP/DOXP pathway (1-deoxy-D-xylulose-5-phosphate reductoisomerase (DXR), 2-C-methyl-D-erythritol 4-phosphate cytidylyltransferase (ISPD), 4-diphosphocytidyl-2-C-methyl-D-erythritol kinase (ISPE), 2-C-methyl-D-erythritol 2,4-cyclodiphosphate synthase (ISPF), and 4-hydroxy-3-methylbut-2-en-1-yl diphosphate reductase (ISPH) had the same copy number in *A. sinensis*, grape and cacao) is relatively stable.

By screening event-related alignments within the species, we obtained the number of sesquiterpene synthesis genes related to different events. In the entire pathway regulating sesquiterpene synthesis of *A. sinensis*, 64.1 % (25/39) of genes are related to ECH event, and 51.3 % (20/39) of genes are related to AST event (Additional file 1: Table S17). Especially, there are five 1-deoxy-D-xylulose 5-phosphate synthase (DXS) genes (the rate-limiting enzyme of the MEP/DOXP pathway [27–29]) in *A. sinensis*. Genome synteny analysis showed that *As3g00334* and *As1g01631* fall into two large syntenic blocks on *A. sinensis* chr3 and chr1 that correspond to a homologous block on grape chr5 and cacao chr4 (Additional file 2: Figure S9), indicating that the *As3g00334* and *As1g01631* might be a product of the AST event.

By analyzing the transcriptome data of the *A. sinensis*, we found that the expression of most of genes involved in the regulatory pathways increased after injury, such as *As7g01288* (ACAT in MVA pathway), *As4g02461* (geranylgeranyl diphosphate synthase (GGPS)) and *As4g00176* ((3 S,6E)-nerolidol synthase (NES1)) (Fig. 5, Additional file 1: Table S18). It’s interesting that the expression profiles of some homologues of genes are different. For example, the five homologs of the DXS gene have four types of expression profiles, especially the expression of *As3g00334* increased after 5 days of injury. In contrast, the expression of *As1g01631* remained unchanged.

Furthermore, except for the gene of NAD+-dependent farnesol dehydrogenase (FLDH), the expression of sesquiterpene synthesis genes was increased in the injured stems. By constructing a phylogenetic tree of the 30 sesquiterpene synthesis genes in the three species (Additional file 2: Figure S10), we found that only the topological structure of the FLDH gene is consistent with the species phylogeny (Fig. 2a). However, the topological structures formed by the other four types of genes (GERD, NES1, alpha-farnesene synthase (AFS1), and (+)-delta-cadinene synthase (CDN1)) were inconsistent with the species phylogeny, and all of the *A. sinensis* genes were located outside of the two reference species, indicating that the evolution rate of these genes in *A. sinensis* is faster than that of grape and cacao genes. The changes of expression and evolutionary patterns of these genes indicate that they may have specific functions in the process of sesquiterpene synthesis.

## Discussion

### Tetraploidization of ***A. sinensis*** Genome

Recursive polyploidization, ploidy-different polyploidization, and genome rearrangement events made the structure of plant genomes become complicated [9, 10], which hinders our understanding of the evolution of the genome and the function of genes. Although the genome size could not indicate whether it has experienced polyploidization, the genome sizes of species can be used as a rough estimate for the existence of WGD. The genome size of *A. sinensis* is ~ 773 Mb, which is 72.2 and 75.3 % larger than grape (~ 449 Mb)[30] and cacao (~ 441 Mb)[31], respectively, indicating that *A. sinensis* have experienced a tetraploidization. A crucial consideration for exploring the multiples of genome-wide polyploidization is to determine the scales of orthologous and paralogous collinear blocks through inter-genome and intra-genome comparisons, respectively. Based on the analysis of the 4DTv distribution of gene pairs in the collinear block, a large accumulation of gene duplications in the *A. sinensis* genome was reported[11]. Ding et al. concluded that the genome of *A. sinensis* seems to have experienced a recent whole-genome duplication event, without any evidence of the ploidy of polyploidization. In the current study, we found the ratios of orthologous regions of grape and cacao with *A. sinensis* are both 1:2, indicating that the polyploidization of *A. sinensis* is a tetraploidization.

In addition, we also generated the gene-colinearity supported homologous (orthologous and paralogous) gene lists, which are helpful to reveal the evolutionary and function-innovation trajectories of genes, gene families, regulatory pathways, and other important traits. For example, based on the lists, we found that genes related to the sesquiterpene biosynthetic pathway are expanded during polyploidization events.

### Evolutionary Rates

Different species have different evolutionary rates, the differences may be even greater especially after being affected by polyploidization. To perform authentic dating, we corrected the evolutionary rate of cacao and *A. sinensis* based on the grape, which has the slowest evolutionary rate. We found that the evolutionary rate of *A. sinensis* is 49.9 and 21.7 % ((1.9379–1.5922)/1.5922) faster than that of grape and cacao, respectively.

### Possible Factors of Sesquiterpene Synthesis in ***A. sinensis***

Previous studies have demonstrated that sesquiterpenes and phenylethyl chromone derivatives are the main compounds in agarwood[32–34], and the terpenoid metabolism pathway is very clear. Polyploidization may promote the evolution of key traits, such as nodulation[35], cotton fiber[26], and VC biosynthesis[36]. In the present study, we found that the genes of *A. sinensis* involved in the sesquiterpene synthesis pathway were also affected by two polyploidization events. Besides, more than half of the identified genes related to sesquiterpene synthesis have experienced AST events. The function of some sesquiterpene synthesis genes related to AST may be different and play distinct roles in the process of sesquiterpene synthesis. Phylogenetic analysis showed that four of the five types of sesquiterpene synthase genes in *A. sinensis* evolved faster than those in the other two species, suggesting their distinct functions in *A. sinensis*. In addition, the expression of genes in the sesquiterpene synthesis regulatory pathway has changed after the injury of *A. sinensis*, and most of the genes are highly expressed in the stem. In particular, two genes (*As3g00334* and *As1g01631*) presented opposite expression patterns, indicating that they may have different functions after the AST event.

## Conclusions

In conclusion, by reanalyzing its genome, we found that *A. sinensis* had experienced a tetraploidization event at ~ 63-71Mya. Further analysis demonstrated that the polyploidization of *A. sinensis* contributed to the functional differentiation of genes related to sesquiterpene synthesis. The present work also provided an alignment of orthologous and paralogous genes related to different events of polyploidization and speciation, which provides an important comparative genomics resource for Thymelaeaceae and related families. We hope that these results will not only be helpful for illustrating the evolution of *A. sinensis*, but also for understanding the functions of genes related to the formation of agarwood.

## Methods

### Genomes and RNA-seq data

Since the grape and cacao didn’t experience recent whole-genome duplication after the core-eudicot-common hexaploidy (ECH), they were selected as reference genomes in the present work. The genomes of grape, cacao and *A. sinensis* together with their annotation files were downloaded from the public databases listed in Table S19. The RNA-seq data of *A. sinensis* stem (intact, wounded 5 days, and wounded 5 + 1 days) generated by Nong et al. was obtained from the NCBI Short Read Archive (SRR10143145, SRR10143137, and SRR10143136)[12].

### Gene Collinearity

The homologous proteins within and between genomes were identified by using the BLASTP[37] (E value 1 × 10^− 5^), and the syntenic blocks consisting of homologous genes were scanned by using the ColinearScan[22]. The maximum gap length between genes that are collinearity along a chromosome was set as 50 genes apart [21, 38]. By using the wgdi package of Python (https://github.com/SunPengChuan/wgdi), we produced dotplots of the homologous gene within or between genomes. All procedures followed the gold-standard pipeline for deciphering complex genomes[20].

### Nucleotide Substitution

Synonymous nucleotide substitutions on synonymous sites (${K}_{S}$) between colinear homologous genes were estimated by using the YN00 program in the PAML (v4.9i) package with the Nei-Gojobori approach[39]. According to their *K*_*S*_ values, we displayed the collinearity gene pairs in the dotplot with different colors, which facilitate to cluster the blocks generated by different events into groups.

### Construction of An Event-Related, Colinear Gene Table

To construct the polyploid event-related colinear gene table with the grape genome as a reference, we listed all grape genes in the first column. Each grape gene may have two extra colinear genes due to the hexaploidy of the genome. Thus, we assigned the extra colinear genes into the other two columns in the table. For each gene from the grape, when there was a corresponding colinear gene in an expected location, we entered its ID in a cell of the corresponding column in the table. When a colinear gene was missing, often due to gene loss or translocation in the genome, we entered a dot in the cell. For the cacao genome, except for sharing hexaploidization with grape, it has no extra duplications. Therefore, we assigned one column next to the columns of grape. Meanwhile, for the *A. sinensis* genome that experienced a paleo-tetraploidization event (ref. the analysis in the Result section), we assigned two columns in the table. Finally, a table with 12 columns was built, which reflects layers and layers of threefold and then twofold homology due to recursive polyploidies across the genomes. The cacao-based table was constructed similarly.

### Kernel Function Analysis of *K*_*s*_

To exhibit the enrichment of ${K}_{S}$, we performed a kernel function analysis of the ${K}_{S}$ distribution of collinear genes within a genome or between genomes. The Gaussian_kde function in the scipy package of Python was used to estimate the probability density of each ${K}_{S}$ list, and to obtain the density distribution curve. Subsequently, the curve_fit function was used to obtain the proper curves by adjusting related parameters with the Gaussian equation. We adjusted the R^2^ to be as close to 1 as possible, and finally determined the goodness of the fit.

### KEGG Annotation

Assigning KEGG Orthologies (KO) to the predicted proteins of the three genomes and generating the KEGG metabolic pathways were done with KAAS (KEGG Automatic Annotation Server)[40] using the bi-directional best hit method.

### Calculation of Gene Expression Levels

The raw Illumina reads were trimmed by using Trimmomatic (v0.39)[41]. The FastQC[42] qualified reads were aligned to the *A. sinensis* genome by using the hierarchical indexing for spliced alignment of transcripts (HISAT2)[43]. The aligned results were sorted and transferred the format from ‘bam’ to ‘sam’ by using SAMtools[44], and the transcripts per kilobase of exon model per million mapped reads (TPM) for each *A. sinensis* gene model was calculated by using Stringtie [45].

### Construction of Phylogenetic Tree

The peptide sequences (PEP) of the sesquiterpenoid biosynthesis genes in three genomes were aligned by using ClustalW[46, 47]. The tree construction was performed by using the neighboring-joining approach in MEGA X[48] with default parameters. The reliability of an inferred tree was characterized with bootstrap analysis with 1000 replications.

## Supplementary Information

**Additional file 1:** **Table S1**. Number of Homologous Blocks and Gene Pairs Within a Genome or Between Genomes. **Table S2**. Number of Homologous Genes Within a Genome or Between Genomes. **Table S3**. Information about the Blocks Related to ECH Events in *A. sinensis* genome. **Table S4**. Information about the Blocks Related to AST Events in *A. sinensis* genome. **Table S5**. Information about the Blocks Related to ECH Events in Grape Genome. **Table S6**. Information about the Blocks Related to ECH Events in Cacao Genome. **Table S7**. Number of Collinearity Blocks, Gene Pairs, and Genes Related to Species Divergence Events Between Genomes. **Table S8**. Multiple Genome Alignment with Grape as Reference. **Table S9**. Genome Alignment Between Cacao and *A.sinensis.***Table S10**. *A. sinensis* Gene Loss Rates and Gene Translocation with Grape as Reference Genome. **Table S11**. *A. sinensis* Gene Loss Rates and Gene Translocation with Cacao as Reference Genome. **Table S12**. Gene Loss in *A. sinensis* Using Grape and Cacao as References. **Table S13**. The Observed Distribution of Gene Loss and Translocation Numbers Fitted by Using Different Density Curves of Geometry Distribution. **Table S14**. Kernel Function Analysis of ${K}_{S}$ Distribution Related to Duplication Events Within each Genome and Between Genomes (before Evolutionary Rate Correction). **Table S15**. Kernel Function Analysis of ${K}_{S}$ Distribution Related to Duplication Events Within each Genome and Between Genomes (after Evolutionary Rate Correction). **Table S16**. The Information of Terpenoid Backbone Biosynthesis and Sesquiterpenoid Biosynthesis Genes in the Three Genomes by KEGG. **Table S17**. Sesquiterpenoid Biosynthesis Genes. **Table S18**. The Expression Values (TPM) of *A. sinensis* stems in Three States (intact, wounded 5 days and wounded 5 + 1 days) by RNA-sEq. **Table S19**. Genomic Data Information.

**Additional file 2:** **Figure S1**. Homologous Genes Dotplot between *A. sinensis* and Grape Genomes. **Figure S2**. Homologous Genes Dotplot between *A. sinensis* and Cacao Genomes. **Figure S3**. Local Homologous Alignments of Grape, Cacao, and *A. sinensis* Genomes. **Figure S4**. The Retention of Duplicated Genes Residing in Two Subgenomes of *A. sinensis* using the Grape as Reference. **Figure S5**. The Retention of Duplicated Genes Residing in Two Subgenomes of *A. sinensis* using the Cacao as Reference. **Figure S6**. Near Geometric Distribution of Continually Lost or Translocated Genes between *A. sinensis* and Grape. **Figure S7**. Near Geometric Distribution of Continually Lost or Translocated Genes between *A. sinensis* and Cacao. **Figure S8**. Histograms and Gaussian Fitted Curves of ${K}_{S}$ between Colinear Homologous Genes. **Figure S9**. Gene Synteny Analysis among Grape, Cacao, and *A. sinensis*. **Figure S10**. The Phylogenetic Tree Constructed using Sesquiterpene Synthesis Genes from Grape, Cacao, and *A. sinensis*.

## Data Availability

All data generated or analysed during this study were included in this published article and the Additional files.

## Abbreviations

WGD: Whole-genome duplication; As:*Aquilaria sinensis*
Vv: *Vitis vinifera*
Tc: *Theobroma cacao*
MVA: Mevalonic acid
DOXP: 1-deoxy-D-xylulose-5-phosphate
MEP: Methylerythritol phosphate
ECH: Core Eudicot-Common Hexaploidy
Mya: Million years ago
*K*_*S*_: Synonymous nucleotide substitutions on synonymous sites
AST: *A. sinensis*-specific tetraploidization
ACAT: Acetyl-CoA C-acetyltransferase
HMGS: Hydroxymethylglutaryl-CoA synthase
HNGCR: Hydroxymethylglutaryl-CoA reductase
mvaK1: Mevalonate kinase
mvaK2: Phosphomevalonate kinase
MVD: Diphosphomevalonate decarboxylase
DXS: 1-deoxy-D-xylulose-5-phosphate synthase
DXR: 1-deoxy-D-xylulose-5-phosphate reductoisomerase
ISPD: 2-C-methyl-D-erythritol 4-phosphate cytidylyltransferase
ISPE: 4-diphosphocytidyl-2-C-methyl-D-erythritol kinase
ISPF: 2-C-methyl-D-erythritol 2,4-cyclodiphosphate synthase
ISPG: (E)-4-hydroxy-3-methylbut-2-enyl-diphosphate synthase
ISPH: 4-hydroxy-3-methylbut-2-en-1-yl diphosphate reductase
IDI: Isopentenyl-diphosphate Delta-isomerase
GPS: Geranyl diphosphate synthase
FDPS: Farnesyl diphosphate synthase
GGPS: Geranylgeranyl diphosphate synthase, type III
FLDH: NAD+-dependent farnesol dehydrogenase
AFS1: Alpha-farnesene synthase
NES1: (3 S,6E)-nerolidol synthase
CDN1: (+)-delta-cadinene synthase
GERD: (-)-germacrene D synthase
