# Supplementary material for: A tetraploidization event shaped the Aquilaria sinensis genome and contributed to the ability of sesquiterpenes synthesis
Source: BMC Genomics. 2021 Sep 7;22:647. doi: 10.1186/s12864-021-07965-9 (PMC8424979; doi:10.1186/s12864-021-07965-9)
Supplement: Supplementary file 2 — Additional file 2: Figure S1. Homologous Genes Dotplot between A. sinensis and Grape Genomes. Figure S2. Homologous Genes Dotplot between A. sinensis and Cacao Genomes. Figure S3. Local Homologous Alignments of Grape, Cacao, and A. sinensis Genomes. Figure S4. The Retention of Duplicated Genes Residing in Two Subgenomes of A. sinensis using the Grape as Reference. Figure S5. The Retention of Duplicated Genes Residing in Two Subgenomes of A. sinensis using the Cacao as Reference. Figure S6. Near Geometric Distribution of Continually Lost or Translocated Genes between A. sinensis and Grape. Figure S7. Near Geometric Distribution of Continually Lost or Translocated Genes between A. sinensis and Cacao. Figure S8. Histograms and Gaussian Fitted Curves of ${K}_{S}$KS between Colinear Homologous Genes. Figure S9. Gene Synteny Analysis among Grape, Cacao, and A. sinensis. Figure S10. The Phylogenetic Tree Constructed using Sesquiterpene Synthesis Genes from Grape, Cacao, and A. sinensis. [file 12864_2021_7965_MOESM2_ESM.docx]

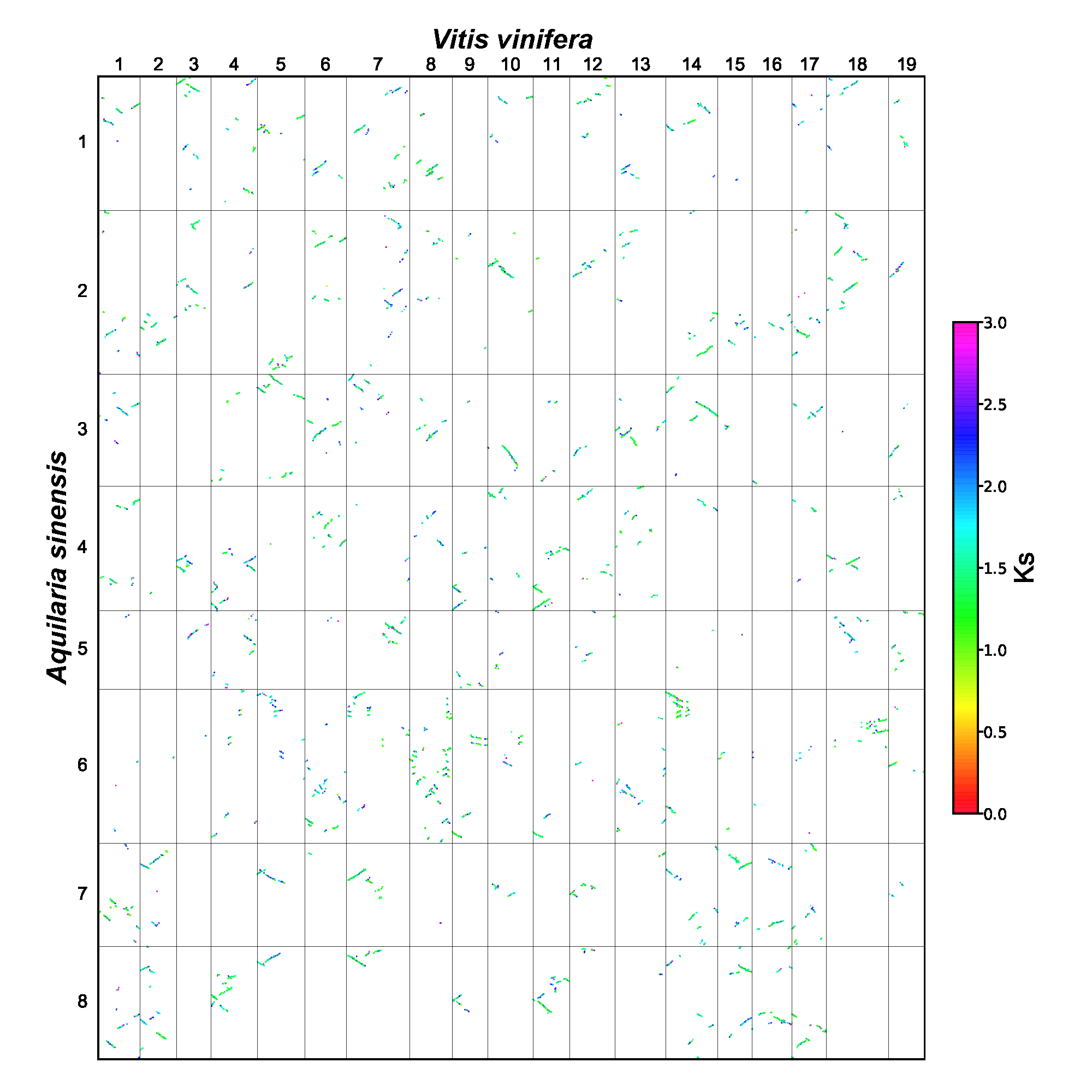


**Figure S1. Homologous Genes Dotplot between *A. sinensis* and Grape Genomes.** $K_{S}$ value for homologous genes on each inferred colinear block is shown, in color from red to purple as to the value of $K_{S}$.


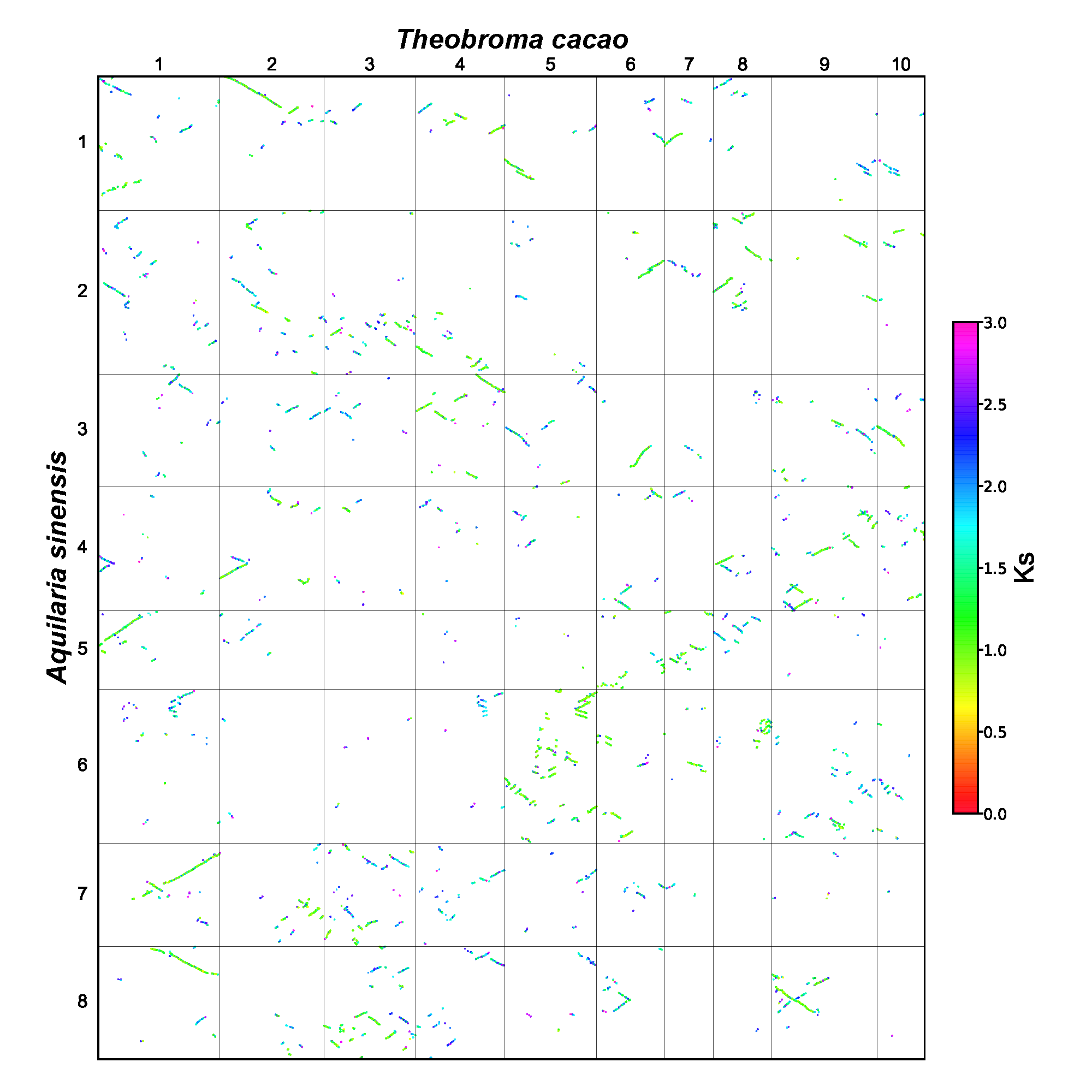


**Figure S2. Homologous Genes Dotplot between *A. sinensis* and Cacao Genomes.** $K_{S}$ value for homologous genes on each inferred colinear block is shown, in color from red to purple as to the value of $K_{S}$.


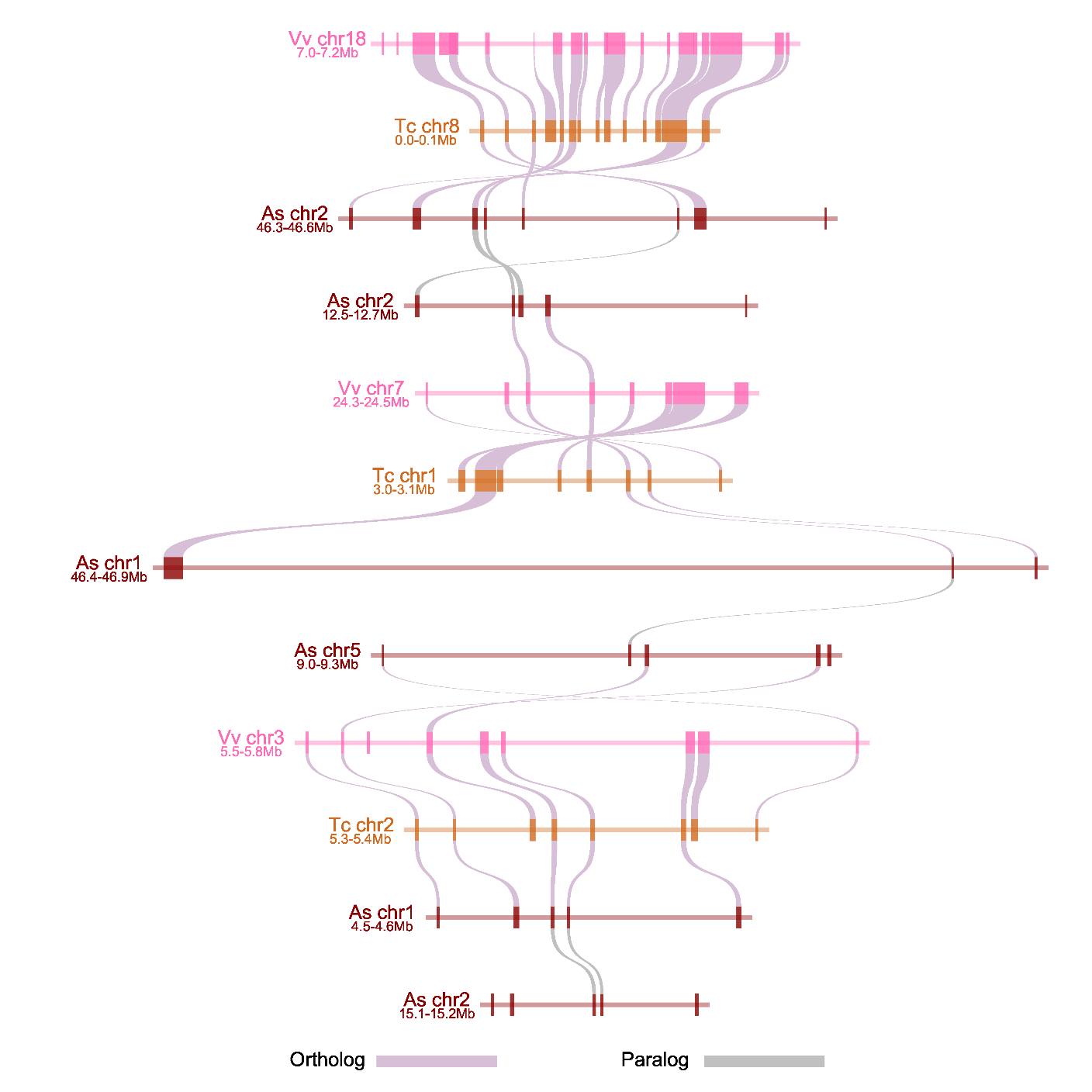


**Figure S3. Local Homologous Alignments of Grape, Cacao, and *A. sinensis* Genomes.** Species are shown in different colors; homologous genes between adjacent chromosomes are ligated with Bezier curves.


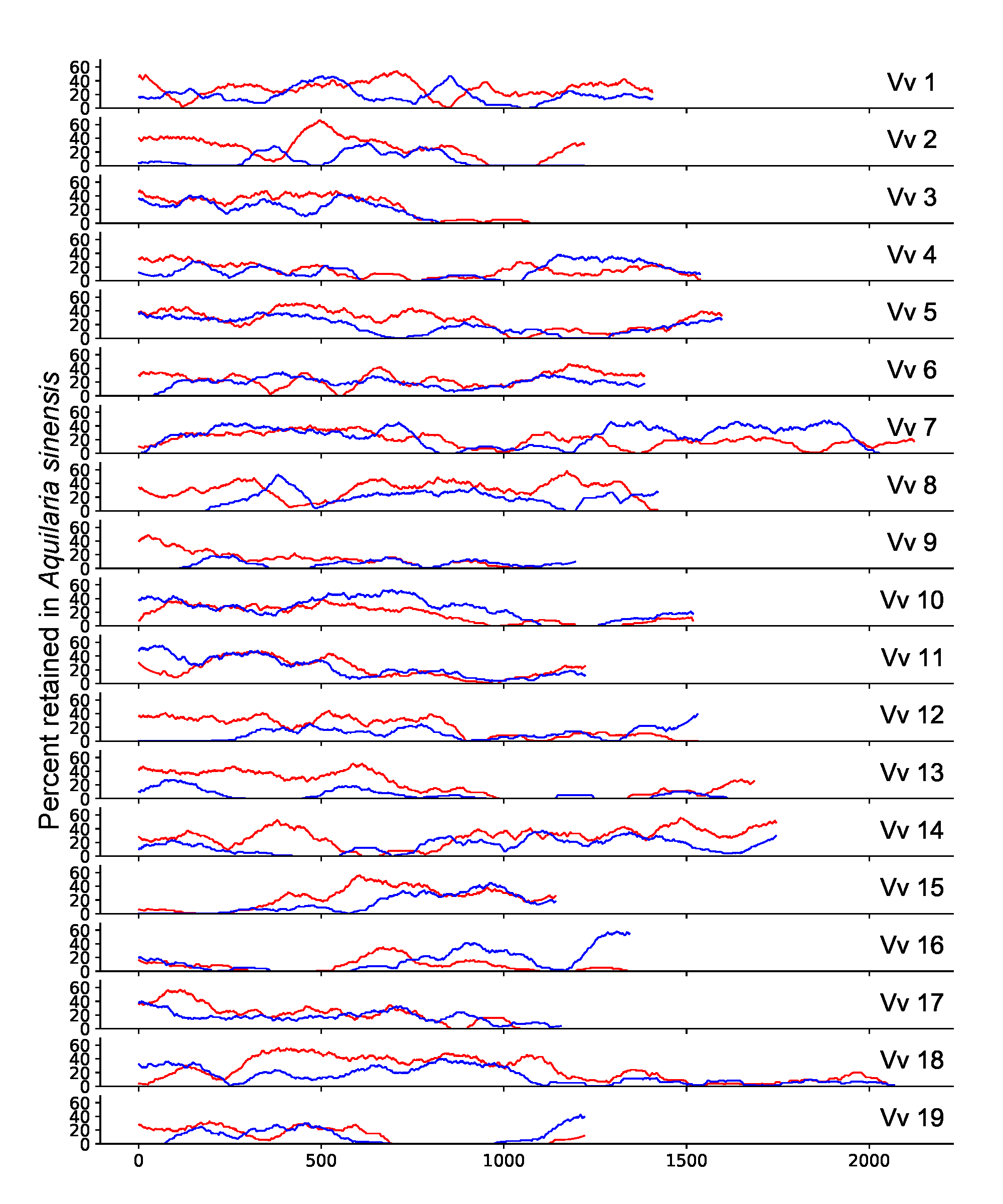


**Figure S4. The Retention of Duplicated Genes Residing in Two Subgenomes of** ***A. sinensis* using the Grape as Reference.** The x-axis represents the number of genes on chromosomes of the grape genome, and the y-axis represents the percentage of genes retained in the *A. sinensis* genomes. The percentages of genes retained of the two subgenomes in the sliding window are represented by red and blue lines, respectively.


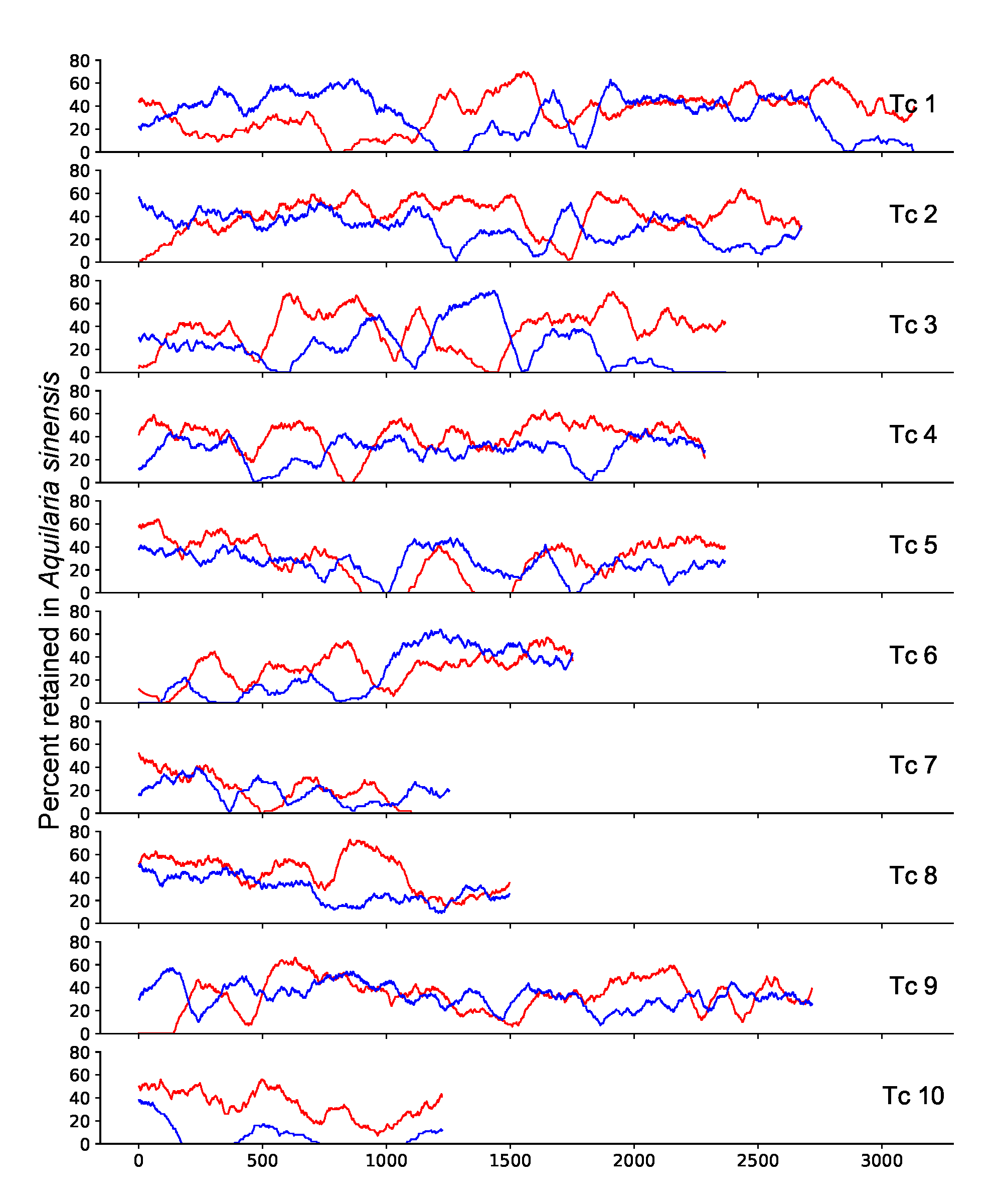


**Figure S5. The Retention of Duplicated Genes Residing in Two Subgenomes of *A. sinensis* using the Cacao as Reference.** The x-axis represents the number of genes on chromosomes of the cacao genome, and the y-axis represents the percentage of genes retained in the *A. sinensis* genomes. The percentages of genes retained of the two subgenomes in the sliding window are represented by red and blue lines, respectively.


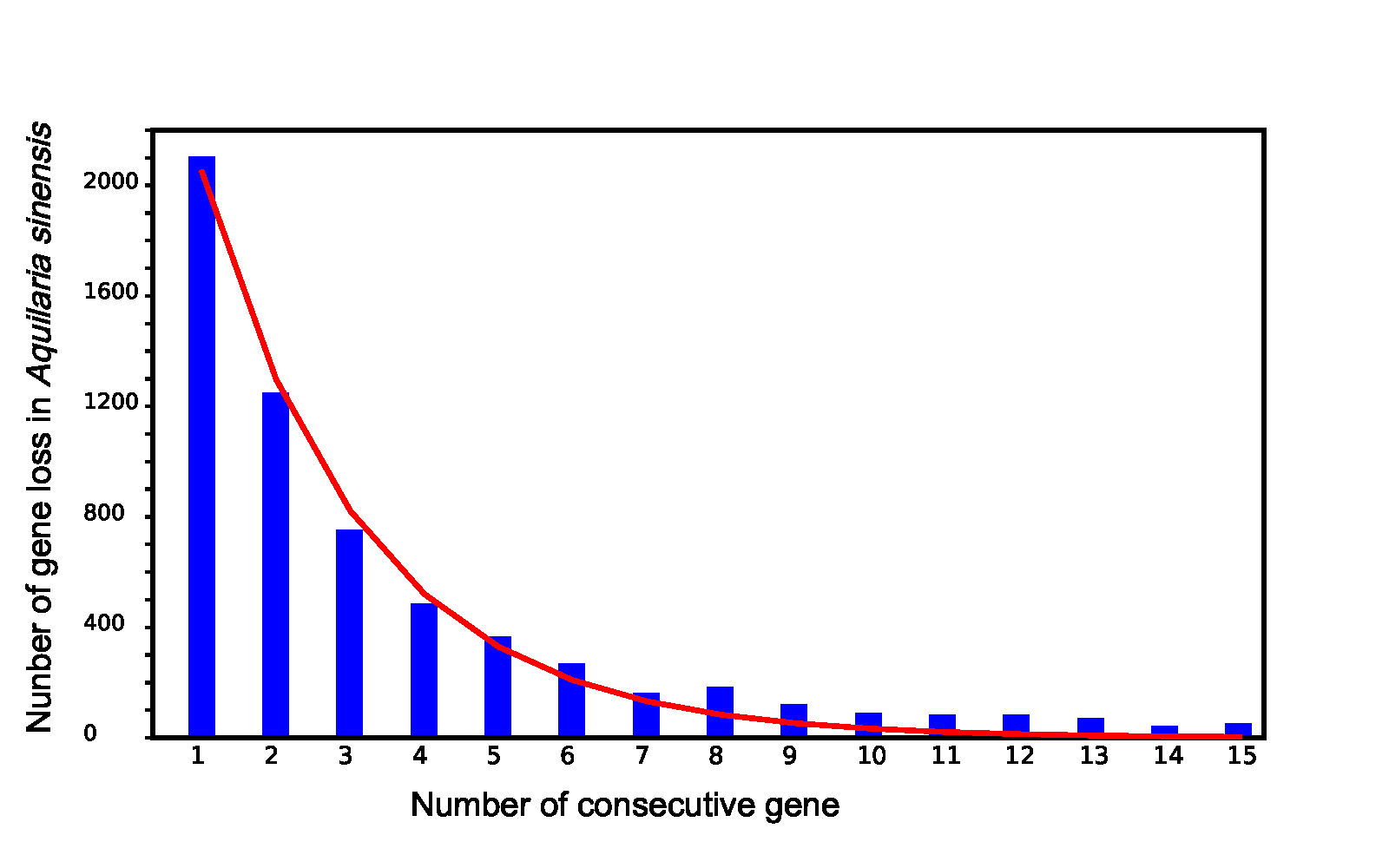


**Figure S6.** **Near Geometric Distribution of Continually Lost or Translocated Genes between *A. sinensis* and Grape.** The x-axis represents numbers of continually lost or translocated genes in the inferred homologous regions. The y-axis represents the number of removed genes of *A. sinensis*.


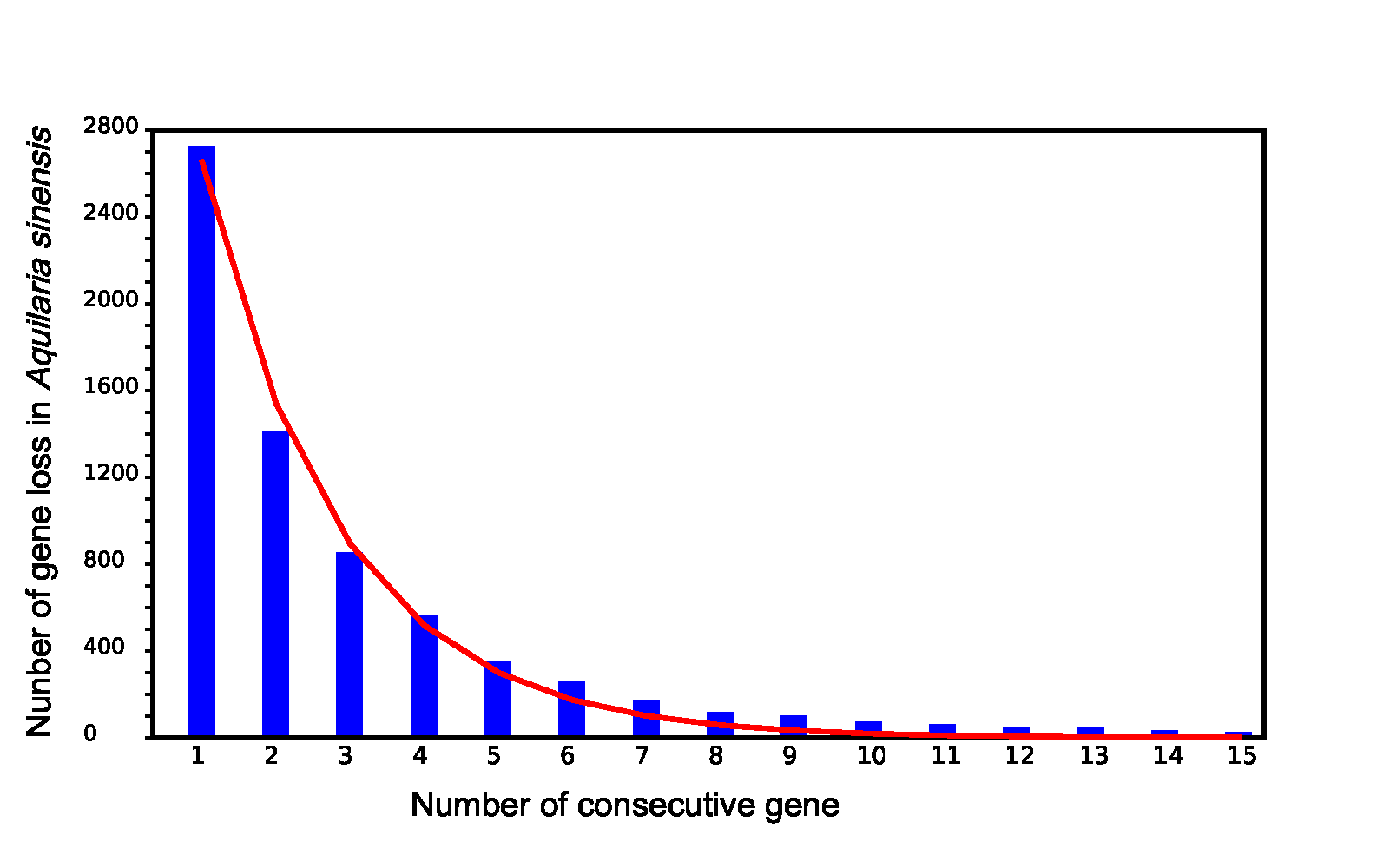


**Figure S7. Near Geometric Distribution of Continually Lost or Translocated Genes between *A. sinensis* and Cacao.** The x-axis represents numbers of continually lost or translocated genes in the inferred homologous regions. The y-axis represents the number of removed genes of *A. sinensis*.


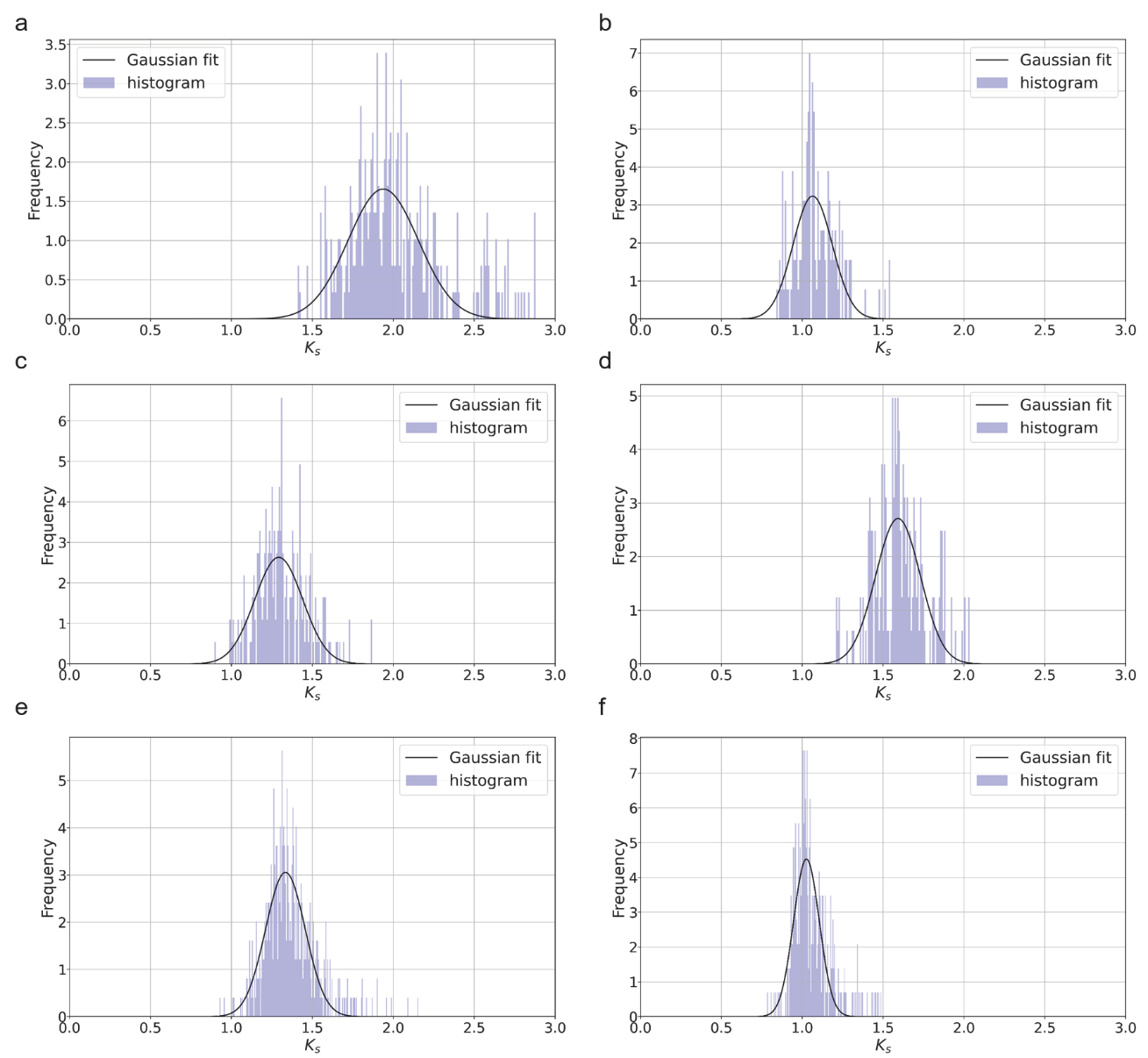


**Figure S8. Histograms and Gaussian Fitted Curves of** $\boldsymbol{K}_{\boldsymbol{S}}$ **between Colinear**

**Homologous Genes.** The median $K_{S}$value of each homologous block is used. a ECH-related homologous blocks in *A. sinensis*; b AST-related homologous blocks in *A. sinensis*; c ECH-related homologous blocks in grape; d ECH-related homologous blocks in cacao; e Homologous blocks between *A. sinensis* and grape; f Homologous blocks between *A. sinensis* and cacao.


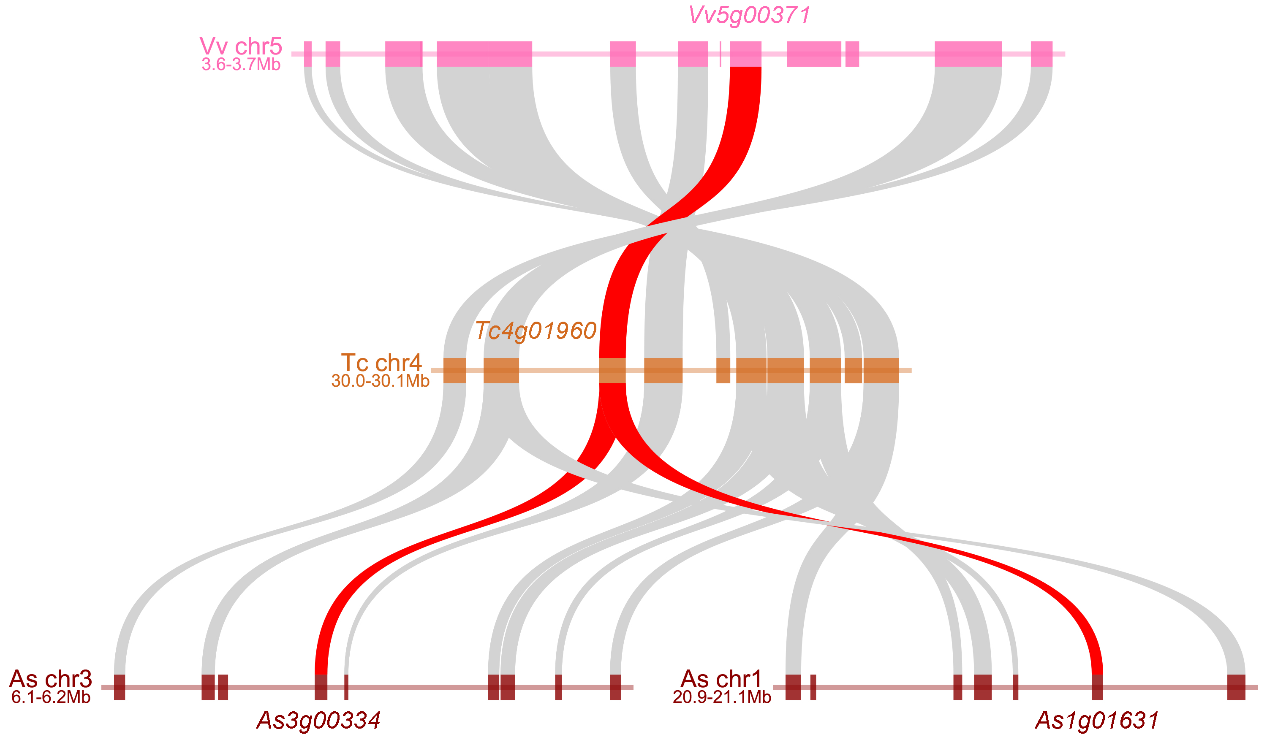


**Figure S9. Gene Synteny Analysis among Grape, Cacao, and *A. sinensis*.** The syntenic block with *vv5g00366* in Grape seems to correspond to one block with *tc4g01966* in cacao and two blocks containing *as3g00339* and *as1g01627* in *A. sinensis*.


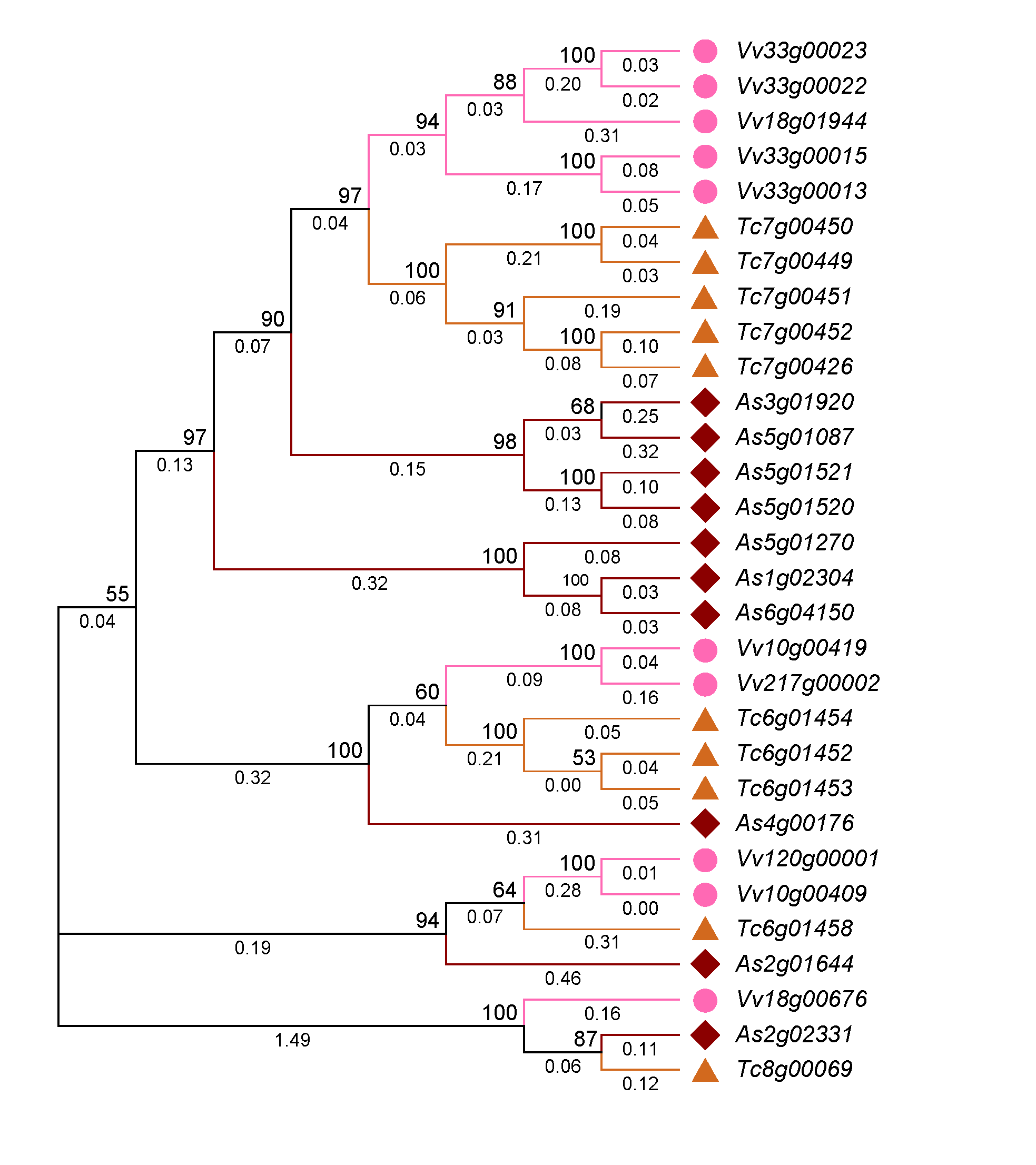


**Figure S10. The Phylogenetic Tree Constructed using Sesquiterpene Synthesis Genes from Grape, Cacao, and *A. sinensis*.** The pink circle represents the grape gene, the chocolate triangle represents the cacao gene, and the dark red diamond represents the *A. sinensis* gene. Bootstrap value is displayed above the branch, and the length of the branch is displayed below the branch.
